# Impact of Red Pack Cell Transfusion Before or After Endoscopy on Mortality in Patients with Upper Gastrointestinal Bleeding: A Multicenter Cohort Study

**DOI:** 10.3390/diseases13100329

**Published:** 2025-10-04

**Authors:** Clelia Marmo, Cristina Bucci, Marco Soncini, Maria Elena Riccioni, Riccardo Marmo

**Affiliations:** 1 CEMAD Centro Malattie Dell’Apparato Digerente, Fondazione Policlinico Universitario Agostino Gemelli, IRCCS, Università Cattolica del Sacro Cuore, Largo Agostino Gemelli, 00168 Rome, Italy; 2 Gastroenterology and Hepatology Unit, AORN Santobono-Pausilipon, 80122 Napoli, Italy; c.bucci@santobonopausilipon.it; 3 Department of Internal Medicine, “A. Manzoni” Hospital, 23900 Lecco, Italy; ma.soncini@asst-lecco.it; 4 Digestive Endoscopy Unit, Fondazione Policlinico Universitario Agostino Gemelli, IRCCS, Università Cattolica del Sacro Cuore, Largo Agostino Gemelli, 00168 Rome, Italy; mariaelena.riccioni@policlinicogemelli.it; 5 Gastroenterology and Endoscopy Unit, “L. Curto” Hospital, ASL Salerno, 84035 Polla, Italy

**Keywords:** upper gastrointestinal bleeding, transfusion strategy, timing for transfusion, mortality

## Abstract

Background: The impact of transfusion timing relative to endoscopy in upper gastrointestinal bleeding (UGIB) remains unclear. Aim: To assess whether transfusion performed before versus after endoscopy affects 30-day mortality in UGIB. Methods: We conducted a post hoc analysis of a multicenter cohort study including 3324 UGIB patients consecutively admitted in hospital. Propensity score matching adjusted for clinical and procedural variables. Results: Among 2017 transfused patients, 34.7% received Red Blood Cells (RBC) before endoscopy. Patients who received transfusions before endoscopy were older, had more severe comorbidities, and presented with a worse physical and hemodynamic status. This study also explored whether transfusion timing relative to endoscopy affects clinical outcomes in patients stratified by baseline hemoglobin levels. While pre-endoscopy transfusion was not significantly associated with reduced 30-day mortality in the overall population, we observed an advantage in patients transfused before the endoscopy when the Hb value was <7 g/dL. Pre-endoscopy transfusion was associated with a 6% absolute reduction in 30-day mortality (*p* < 0.06), with a greater benefit observed in patients with Hb < 7 g/dL (−27%) and <8 g/dL (−21%). Moreover, for this group of patients more favorable outcome was observed when the endoscopy was performed between 6 and 12 h from admission. These findings suggest that transfusion timing should be integrated into individualized UGIB management and may impact future clinical guidelines. Conclusions: In patients with severe anemia and UGIB, transfusion before endoscopy may reduce mortality. Timing to transfusion should be considered alongside hemodynamic and procedural factors in future guidelines.

## 1. Introduction

Upper gastrointestinal bleeding (UGIB) remains a prevalent and potentially life-threatening medical emergency, associated with significant morbidity and mortality. The cornerstone of effective management involves timely resuscitation, diagnostic endoscopy, and appropriate transfusion strategies. While several studies have evaluated the optimal timing for endoscopic intervention—highlighting its role in risk stratification and hemostatic therapy—the influence of transfusion timing on patient outcomes is less defined [1,2,3].

Recent studies have emphasized the potential clinical importance of transfusion timing in patients with upper gastrointestinal bleeding, non-variceal sources of bleeding, and hemoglobin levels below 7.5 g/dL [4].

Guo et al., in a territory-wide cohort study of 6474 patients with acute upper gastrointestinal bleeding, assessed the impact of endoscopy timing. Their findings indicate that patients undergoing endoscopy within 6 to 24 h of admission had more favorable outcomes compared to those receiving urgent (<6 h) or delayed (24–48 h) endoscopy [5]. Specifically, urgent endoscopy was linked with higher 30-day mortality (adjusted HR 1.43) and intensive care unit (ICU) admission rates, potentially due to insufficient time for resuscitation and medical optimization prior to the procedure. These data support current guideline recommendations to perform endoscopy within 24 h but also underline the importance of an adequate pre-endoscopic preparation. European guidelines emphasize that, following the initial clinical assessment, hemodynamic stabilization should be undertaken when necessary, typically through fluid resuscitation with crystalloids or saline solutions [2].

In this context, despite current guidelines addressing transfusion strategies in both variceal and non-variceal gastrointestinal bleeding [1,2], they do not provide recommendations regarding the timing of transfusion in relation to the performance of endoscopy. Previous studies recommended a restrictive transfusion strategy for most UGIB patients (7–9 g/dL hemoglobin) [6,7]. So far, only one study has directly compared the outcomes of transfusion initiated before versus after endoscopic procedures [4]. Additionally, the procedure itself may pose physiologic challenges, including transient hypoxemia, particularly in patients with underlying anemia [8,9].

Given the paucity of direct evidence on transfusion timing relative to endoscopy, we conducted the present study to assess whether initiating transfusion prior to versus after upper gastrointestinal endoscopy is associated with differences in 30-day mortality among patients with UGIB. We hypothesized that identifying a potential impact of timing for transfusions could refine transfusion strategy and contribute to the development of time-sensitive therapeutic algorithms.

## 2. Materials and Methods

This study was a post hoc analysis of a prospective multicenter nationwide cohort analysis conducted across 50 Italian hospitals under the GISED Study Group database (Gruppo Italiano Studio Emorragia Digestiva) that prospectively collected records on all consecutive patients admitted for acute UGIB from 1 January 2014 to 31 December 2015.

During the study period all consecutive patients with overt and ongoing UGIB underwent endoscopy to confirm site and source of bleeding. Exclusion criteria included low/intermediate gastrointestinal bleeding and non-consent for data collection. Patients were defined as having upper gastrointestinal bleeding if they exhibited clinical evidence of overt bleeding upon admission or reported hematemesis, coffee-ground vomiting, melena, hematochezia, or any combination thereof within the 24 h preceding admission. Following initial clinical evaluation—including assessment of hemodynamic status, rectal examination, complete blood count, and coagulation parameters—patients underwent upper gastrointestinal endoscopy within 24 h to identify the source of bleeding. Variceal bleeding and non-variceal bleeding were classified and treated according to guidelines [1,2]. Baseline clinical and endoscopic data were systematically collected, including demographic information (age, sex, site, and date of endoscopy), medical history (presence of comorbidities and medications prescribed prior to the bleeding event), and relevant clinical parameters (hemodynamic status, laboratory values, and other pertinent clinical findings). Endoscopic reports were reviewed for lesion characteristics (location, size, and stigmata of bleeding), type of endoscopic hemostasis performed, and the use of medications before and after endoscopy, including H2-receptor antagonists, tranexamic acid, and proton pump inhibitors.

Additional data collected included episodes of rebleeding, the number of transfusions administered during hospitalization, length of hospital stay, failure of endoscopic hemostatic therapy, requirement for surgical or radiological interventions, and mortality. To assess the relationship between hemoglobin (Hb) levels, transfusion practices, and clinical outcomes, patients were stratified into five groups based on Hb levels at presentation and after red blood cell transfusion: (a) ≤7 g/dL, (b) 7–8 g/dL, (c) 8–9 g/dL, (d) 9–10 g/dL, and (e) >10 g/dL. All participating centers followed transfusion strategies approved according to their local institutional protocols.

Transfusion timings were categorized as “pre-endoscopy” when initiated before the start of an endoscopy and “post-endoscopy” when initiated after or during the endoscopic procedure.

Time to endoscopy was stratified into: ≤6 h, 6–12 h, 12–24 h, and ≥24 h from emergency department presentation according to ESGE guidelines [2]. Re-bleeding was defined as the recurrence of hematemesis, melena, or hematochezia accompanied by hemodynamic instability or as a drop in hemoglobin level ≥ 2 g/dL, following an initial period of clinical stabilization and/or successful endoscopic hemostasis [1]. Shock index was defined according to Rai A et al. [10]. Severity scores were collected to assess patient risk and included ASA physical status classification [11,12], AIMS65 score [13], Glasgow–Blatchford score [14], and ABC score [15].

Mortality was classified as bleeding-related if occurring within 30 days for non-variceal bleeding or within 42 days for variceal bleeding [1,2,3,4,5,6,7,8,9,10,11,12,13,14,15,16]. Secondary outcomes included hospital length of stay, rebleeding events, need for surgery or interventional radiology, and mortality.

### 2.1. Ethics

The Institutional Review Board approved the study protocol in each participating center following the Ethical Committee’s approval (N. 556, 26 June 2013; ‘San Carlo Borromeo’ Hospital, Milan). Written informed consent was obtained from all patients or their healthcare proxies.

### 2.2. Statistics

Sample size calculation was estimated at 1734 patients to detect a between-group difference of 3.6% in mortality, assuming as reference 7.1% [17], considering 90% power, alfa error of 5% and an unequal group size allocation ratio = 0.3. Descriptive statistics were used to summarize patient characteristics. Continuous variables were expressed as means ± standard deviations or medians with interquartile ranges and compared using Student’s *t*-test or Mann-Whitney U test. Categorical variables were compared using chi-square or Fisher’s exact test as appropriate. To minimize potential selection bias and confounding factors, the propensity score matching (PSM) was employed. Patients in the pre- and post-endoscopy transfusion groups were matched in a 1:1 ratio using nearest-neighbor matching with a caliper of 0.03 standard deviations, without replacement. To further explore the interaction between transfusion timing and clinical outcomes, a stratified analysis by hemoglobin level at admission was performed. The average treatment effect (ATE) was estimated using a regression adjustment model. Covariate balance between groups was assessed using standardized mean differences, with values < 0.1 considered acceptable. Additionally, endoscopy timing categories (<6 h, 6–12 h, >12 h) were incorporated into the final model to evaluate the optimal window for combined hemodynamic and endoscopic management. Missing data were handled by listwise deletion. Statistical analyses were performed using STATA version 18.0 (StataCorp, College Station, TX, USA).

## 3. Results

Of the 3324 patients included in the analysis, 2017 (60.7%) received at least one red blood cell transfusion. Among these, 700 patients (34.7%) were transfused before endoscopy, and 1317 (65.3%) after. The overall mean age was 69.7 ± 14.9 years.

### 3.1. Clinical Characteristics

Patients who received transfusions before endoscopy were older, had more severe comorbidities, and presented with a worse physical and hemodynamic status (Table 1). Creatinine, blood urea nitrogen (BUN), and international normalized ratio (INR) values were also higher in this group (Table 2).

These patients also received a greater number of transfused RBC units: 3.6 ± 2.8 vs. 2.8 ± 1.9 (*p* < 0.001). Endoscopy was more frequently performed after 6 h from emergency department presentation in this group (Table 3).

Overall, clinical and economic outcomes did not significantly differ between the two groups. Specifically, 30-day mortality was 8.3% among patients transfused after endoscopy compared to 9.9% in those transfused before the procedure (*p* < 0.23; Table 4).

### 3.2. Factors Affecting Mortality

In multivariate logistic regression, key factors associated with increased mortality included a higher ASA score, the presence of active malignancy, hematemesis at presentation, re-bleeding during hospitalization, and the total number of RBC units transfused. The shock index, time to endoscopy, and baseline hemoglobin class were also retained in the final model (Table 5).

### 3.3. Timing to Transfusion

**Figure 1:**
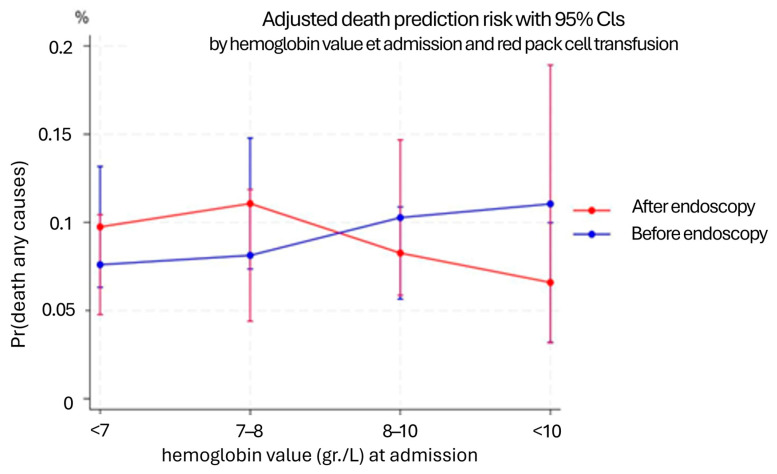
Interaction between hemoglobin value at admission and red pack cell transfusion before or after endoscopy.

The final model also included shock index, timing to endoscopy, and hemoglobin classes at admission, to balance the two groups based on clinically relevant factors for the management of acute gastrointestinal bleeding (Table 5). Stratified analysis showed that among patients with hemoglobin <7 g/dL, those transfused before endoscopy had a 27% relative reduction in mortality (*p* = 0.34). A similar trend was seen in patients with Hb < 8 g/dL (21% reduction, *p* = 0.54). This effect persisted even after adjusting for the source of bleeding in the model (Supplementary Table S1 and Supplementary Figure S1), although not statistically significant, these findings were consistent across models and bleeding sources. The average treatment effect (ATE) for pre-endoscopy transfusion in the Hb < 7 g/dL subgroup was a 6% mortality risk reduction (*p* = 0.06). (Table 6 and Table 7 and Figure 1).

Furthermore, the most pronounced benefit was observed in patients who received transfusions before and underwent endoscopy between 6–12 h after admission. No significant survival advantage was found in patients undergoing endoscopy <6 h or ≥24 h post-admission, highlighting the importance of a time-sensitive, coordinated approach for transfusion and endoscopy. (Supplementary Table S2 and Supplementary Figure S2).

## 4. Discussion

Randomized controlled trials and international guidelines recommend a restrictive transfusion strategy for patients with acute upper gastrointestinal bleeding [1,2,6,7,18,19]. We decided not to differentiate between variceal and non-variceal bleeding, as our objective was to reflect real-world clinical practice, where patients often present with gastrointestinal bleeding before the underlying source has been established.

In general, “restrictive strategy” means that transfusions should be started when the hemoglobin levels fall below 7 or 8 g/dL. Guidelines admit that “one size does not fit all”, and thus the restrictive strategy may not be beneficial in patients with severe comorbidities. However, in general they focus only on patients with cardiovascular disease and neither specify at which level of comorbidity the strategy should become more liberal [20], nor what is the right time to transfuse more frail patients. It is generally accepted that transfusing red blood cells and plasma to address blood loss in upper gastrointestinal bleeding (UGIB) is beneficial for restoring and maintaining tissue oxygenation. The objective of resuscitation is to ensure tissue perfusion through volume restitution, thereby sustaining hemodynamic stability. Our findings indicate that the timing of red blood cell transfusion in patients with UGIB holds significant prognostic implications.

In our study, approximately 35% of patients received a transfusion prior to endoscopy. Despite this, the initial hemoglobin levels may underestimate blood loss due to hemoconcentration; these patients were more likely to present with severe anemia and hemodynamic instability, as indicated by lower baseline hemoglobin levels and higher shock indices. Considering these differences, there is a consistent reduction in mortality associated with pre-endoscopy transfusion in patients with hemoglobin levels below 7 or 8 g/dL, suggesting a potential clinical benefit for the most severely anaemic subgroup. Although the differences are not statistically significant, they hold clinical relevance.

Strong influence factors on death risk such as ASA score, shock index, and the presence of malignancy reinforce the need for individualized transfusion strategy thresholds but also on the optimal timing to transfuse. Recent studies have provided critical insights supporting our findings. For instance, the large territory-wide cohort by Guo et al. [5] demonstrated that endoscopy performed between 6 and 24 h post-admission yielded better outcomes than urgent endoscopy (<6 h), primarily due to the opportunity for resuscitation and stabilization. Similarly, Choi et al. [4] found increased 30-day mortality in patients receiving RBC transfusion after 4 h from emergency admission, especially among those with Hb < 7.5 g/dL and with upper nonvariceal bleeding. In our series the protective effect of transfusions before endoscopy persisted independently from the source of bleeding. The highest level of protection is achieved when the transfusion is administered prior to endoscopic evaluation, optimally within 6 to 12 h from the time of hospital admission. Although the observed mortality differences were not statistically significant, the consistent trend across analyses supports a clinically meaningful association between transfusion timing and patient outcomes, especially in those with severe anemia (Hb < 7 g/dL). These findings are in line with prior reports suggesting that delayed transfusion and urgent endoscopy may be associated with increased mortality risk, potentially due to inadequate resuscitation and suboptimal physiologic conditions at the time of the procedure [4,5].

In fact, the endoscopic procedure itself may transiently compromise respiratory function, particularly in hypoxemic patients or those with advanced comorbidities (8). We believe that our study has some limitations and several strengths. Our study is limited by its retrospective design; there was no predefined treatment protocol for the various causes of acute gastrointestinal bleeding, and there was no common policy for transfusion agreed a priori between the participating centers; and the study was underpowered for subgroup analyses across different hemoglobin groups. On the other side, strengths of our study are the large number of participating hospitals, the fact that both academic and community hospitals were included, and the fact that all patients admitted to the participating hospitals were included in the analysis. We included patients with comorbidities and severe gastrointestinal bleeding.

## 5. Conclusions

Transfusion timing should be considered as a modifiable and time-sensitive intervention with direct implications for patient outcomes, independently from the upper gastrointestinal source of bleeding. Our findings highlight the importance of tailoring transfusion strategies not only according to hemoglobin thresholds but also in relation to the timing of endoscopic or interventional procedures. Incorporating timing into transfusion decision-making could inform the design of future clinical trials and contribute to the refinement of current UGIB guidelines, which to date provide no explicit recommendations on this aspect.

## Figures and Tables

**Table 1:** Demographics and Clinical Characteristics.

Variable	After Endoscopy (*N* = 1317)	Before Endoscopy (*N* = 700)	*p*-Value
Age, years mean (SD)	69.1 (±15.1)	70.8 (±14.6)	0.015
Male	900 (68.3%)	467 (66.7%)	0.46
Inpatients’ bleeding	229 (17.4%)	147 (21.0%)	0.048
ASA Score 1	324 (24.6%)	122 (17.4%)	<0.001
ASA Score 2	460 (34.9%)	222 (31.7%)	–
ASA Score 3	450 (34.2%)	285 (40.7%)	–
ASA Score 4	83 (6.3%)	71 (10.1%)	–
Chronic Renal Failure	173 (13.1%)	132 (18.9%)	<0.001
Stroke	40 (3.0%)	27 (3.9%)	0.33
Cerebrovascular Disease	120 (9.1%)	80 (11.4%)	0.097
Chronic ischemic heart disease	281 (21.3%)	175 (25.0%)	0.056
Chronic Pulmonary Disease	151 (11.5%)	99 (14.1%)	0.082
Neoplasia	221 (16.8%)	131 (18.7%)	0.28
Cirrhosis	309 (23.5%)	130 (18.6%)	0.010
ABC score, mean (SD)	5.1 (2.5)	5.2 (2.4)	0.17
AIMS65 score, mean (SD)	1.6 (1.0)	1.9 (1.0)	<0.001
Hematemesis	576 (43.7%)	273 (39.0%)	0.040
Melena	869 (66.0%)	514 (73.4%)	<0.001
Pulse rate, mean (SD)	90.0 (16.5)	91.9 (17.9)	0.022
Blood pressure, mean (SD)	114.5 (22.5)	110.7 (22.9)	<0.001
Shock index	109 (8.3%)	89 (12.7%)	0.002

Abbreviations: ASA = American Society of Anesthesiologists. SD = standard deviation.

**Table 2:** Laboratory Parameters on Admission.

Variable	After Endoscopy	Before Endoscopy	*p*-Value
Hemoglobin at admission (g/dL), mean (SD)	8.3 (1.9)	7.3 (1.9)	<0.001
<7 g/dL	322 (24.5%)	313 (44.7%)	<0.001
7–8 g/dL	301 (22.9%)	182 (26.0%)	–
8–10 g/dL	474 (36.1%)	157 (22.4%)	–
>10 g/dL	217 (16.5%)	48 (6.9%)	–
Creatinine, mean (SD)	1.4 (1.2)	1.5 (1.3)	0.024
INR, mean (SD)	1.4 (0.9)	1.6 (1.5)	<0.001
Albumin, mean (SD)	3.1 (0.7)	3.1 (0.7)	0.14
Bilirubin, mean (SD)	1.5 (4.7)	1.3 (2.7)	0.21
BUN, mean (SD)	136.4 (260.9)	98.1 (148.7)	0.018

Abbreviations: INR = international normalized ratio; BUN = blood urea nitrogen; SD = standard deviation.

**Table 3:** Endoscopy Timing, Setting, and Source of Bleeding.

Variable	After Endoscopy	Before Endoscopy	*p*-Value
0–6 h	905 (68.7%)	383 (54.7%)	<0.001
6–12 h	196 (14.9%)	133 (19.0%)	
12–24 h	146 (11.1%)	133 (19.0%)	
>24 h	70 (5.3%)	52 (7.4%)	
Normal working hours	662 (50.3%)	383 (54.7%)	0.16
Night shift	225 (17.1%)	105 (15.0%)	
On-call shift	428 (32.5%)	211 (30.2%)	
Weekdays	998 (75.8%)	519 (74.1%)	0.063
Weekend	125 (9.5%)	89 (12.7%)	
Preholiday	196 (14.9%)	92 (13.1%)	
Non-variceal bleeding	1051 (79.8%)	582 (83.1%)	0.069
Variceal bleeding	266 (20.2%)	118 (16.9%)	
Transfusions, mean (SD)	2.8 (1.9)	3.6 (2.8)	<0.001
No endoscopic therapy	425 (32.3%)	259 (37.0%)	0.056
Therapy in non-variceal	626 (47.5%)	323 (46.1%)	
Therapy in variceal	266 (20.2%)	118 (16.9%)	

Abbreviations: 0/6, 6/12 etc. = time intervals in hours from admission to endoscopy.

**Table 4:** Clinical Outcomes.

Variable	After Endoscopy	Before Endoscopy	*p*-Value
Length of stay, days (mean ± SD)	11.3 (±10.1)	11.1 (±9.2)	0.59
Rebleeding	121 (9.2%)	69 (9.9%)	0.62
Interventional radiology	17 (1.3%)	16 (2.3%)	0.094
Surgery	54 (4.1%)	38 (5.4%)	0.17
Overall mortality	109 (8.3%)	69 (9.9%)	0.23

Abbreviations: SD = standard deviation.

**Table 5:** Multivariable Logistic Regression—Independent Predictors of Mortality.

Factor	Odds Ratio	Std. Err.	z	*p* > |z|	95% CI
ASA Score 1 (Ref)	–	–	–	–	–
ASA Score 2	3.07	1.39	2.47	0.013	1.26
ASA Score 3	3.82	1.72	2.98	0.003	1.58
ASA Score 4	14.48	6.86	5.64	0.000	5.72
Hemodynamic shock	1.26	0.35	0.82	0.413	0.73
Timing 0–6 h (Ref)	–	–	–	–	–
Timing 6–12 h	0.77	0.21	0.94	0.346	0.45
Timing 12–24 h	0.86	0.26	0.52	0.604	0.48
Timing > 24 h	1.04	0.39	0.11	0.911	0.50
In-hospital bleeding	2.34	0.49	4.10	0.000	1.56
Chronic renal failure	1.44	0.33	1.60	0.109	0.92
Neoplasia	1.88	0.38	3.14	0.002	1.27
Cirrhosis	1.31	0.28	1.25	0.213	0.86
Hematemesis	1.92	0.40	3.14	0.002	1.28
Transfusion number	1.11	0.03	3.62	0.000	1.05
Admission Hb < 7 g/dL (Ref)	–	–	–	–	–
Hb 7–8 g/dL	1.18	0.29	0.66	0.511	0.73
Hb 8–10 g/dL	1.02	0.25	0.10	0.922	0.64
Hb > 10 g/dL	0.84	0.27	0.55	0.586	0.45
Transfusion Before vs. After	0.94	0.18	0.33	0.742	0.64

Abbreviations: CI = confidence interval; ASA = American Society of Anesthesiologists.

**Table 6:** Interaction Between Hemoglobin Level and Endoscopy Timing on Mortality Risk.

Group	Odds Ratio	Std. Err.	z	*p* > |z|	95% CI
<7 g/dL—Before	0.73	0.24	0.96	0.338	0.38
7–8 g/dL—After	1.18	0.38	0.51	0.610	0.62
7–8 g/dL—Before	0.79	0.30	0.62	0.538	0.38
8–10 g/dL—After	0.81	0.25	0.68	0.497	0.44
8–10 g/dL—Before	1.07	0.39	0.19	0.853	0.52
>10 g/dL—After	0.61	0.24	1.23	0.219	0.28
>10 g/dL—Before	1.18	0.63	0.31	0.760	0.41

Abbreviations: CI = confidence interval.

**Table 7:** Average Treatment Effect (ATE) of Endoscopy Timing on Mortality Across Hemoglobin Strata.

Hb Admission (g/dL)	Coefficient	Std. Err.	z	*p* > |z|	95% CI
≤7 g/dL	−0.06	0.03	−1.86	0.063	−0.11 to 0.00
7–8 g/dL	−0.03	0.04	−0.95	0.342	−0.10 to 0.04
8–10 g/dL	0.02	0.03	0.52	0.605	−0.05 to 0.08

Abbreviations: SE = standard error; ATE = average treatment effect; Hb = hemoglobin; CI = confidence interval.

## Data Availability

The data presented in this study are available on reasonable request from the corresponding author due to legal and ethical reasons.

## Supplementary Materials

The following supporting information can be downloaded at: https://www.mdpi.com/article/10.3390/diseases13100329/s1, Table S1: Multivariable logistic regression adjusted by source of bleeding: independent predictors of mortality after RBC transfusion for acute upper gastrointestinal bleeding; Table S2: Multivariable logistic regression: independent predictors of mortality after RBC transfusion for acute upper gastrointestinal bleeding: interaction of transfusion before or after endoscopy by timing to endoscopy; Figure S1: Adjusted death risk, including source of bleeding, by interaction of hemoglobin value at admission and red pack cell transfusion before or after endoscopy; Figure S2. Adjusted death risk, including source of bleeding, by interaction of red pack cell transfusion before or after endoscopy and timing to endoscopy.

## Abbreviations

UGIB	Upper Gastrointestinal Bleeding
RBC	Red Blood Cell
Hb	Hemoglobin
PSM	Propensity Score Matching
ASA	American Society of Anesthesiologists
BUN	Blood Urea Nitrogen
INR	International Normalized Ratio
ESGE	European Society of Gastrointestinal Endoscopy
ACG	American College of Gastroenterology
ASGE	American Society for Gastrointestinal Endoscopy
NICE	National Institute for Health and Care Excellence
BSG	British Society of Gastroenterology
ATE	Average Treatment Effect
ICU	Intensive Care Unit
GBS	Glasgow–Blatchford Score
AIMS65	Albumin, INR, Mental Status, Systolic BP < 90 mmHg, Age > 65
ABC	Age, Blood tests, Comorbidities
IQR	Interquartile Range
SD	Standard Deviation
