# Supplementary material for: Impact of Red Pack Cell Transfusion Before or After Endoscopy on Mortality in Patients with Upper Gastrointestinal Bleeding: A Multicenter Cohort Study
Source: Diseases. 2025 Oct 4;13(10):329. doi: 10.3390/diseases13100329 (PMC12563327; doi:10.3390/diseases13100329)

Supplementary materials

Supplementary Table S1. Multivariable logistic regression adjusted by source of bleeding: independent predictors of mortality after RBC transfusion for acute upper gastrointestinal bleeding

Death any cause	Odds ratio	Std. err.	z	P>|z|	[95% conf.	interval]
ASA score						
1	Reference					
2	3.05	1.38	2.46	0.014	1.25	7.41
3	3.82	1.72	2.98	0.003	1.58	9.23
4	14.38	6.81	5.63	0.000	5.69	36.37
Hemodynamic shock	1.26	0.35	0.85	0.397	0.74	2.17
In Hospital bleeding	2.32	0.48	4.04	0.000	1.54	3.49
Chronic renal failure	1.43	0.32	1.59	0.113	0.92	2.23
Neoplasia	1.90	0.38	3.21	0.001	1.29	2.82
Cirrhosis	1.45	0.42	1.29	0.198	0.82	2.55
Haematemesis	1.94	0.41	3.14	0.002	1.28	2.94
Transfusions nr	1.11	0.03	3.50	0.000	1.05	1.18
Timing to endoscopy, hours						
0/6	Reference					
6/12	0.77	0.21	-0.97	0.334	0.45	1.31
12/24	0.85	0.25	-0.56	0.573	0.47	1.52
>24	1.04	0.39	0.10	0.922	0.50	2.16
Admission Haemoglobin, g/dL						
<7	Reference					
7-8	1.18	0.29	0.66	0.509	0.73	1.90
8-10	1.03	0.25	0.13	0.894	0.64	1.66
>10	0.84	0.27	-0.55	0.583	0.44	1.58
Transfusion Before vs After endoscopy	0.94	0.18	-0.31	0.755	0.64	1.38

Source of bleeding	0.88	0.28	-0.42	0.672	0.47	1.62
_cons	0.00	0.00	-10.50	0.000	0.00	0.00

Supplementary Table S2. Multivariable logistic regression: independent predictors of mortality after RBC transfusion for acute upper gastrointestinal bleeding : interaction of transfusion before or after endoscopy by timing to endoscopy

	Odds ratio	Std. err.	z	P>|z|	[95% conf. interval]	
ASA score						
1	Reference					
2	3.05	1.38	2.46	0.014	1.26	7.42
3	3.80	1.71	2.97	0.003	1.57	9.19
4	14.39	6.81	5.63	0.000	5.69	36.40
Hemodynamic shock	1.25	0.35	0.81	0.418	0.73	2.16
In Hospital bleeding	2.35	0.49	4.09	0.000	1.56	3.54
Chronic renal failure	1.44	0.33	1.61	0.107	0.92	2.25
Neoplasia	1.89	0.38	3.19	0.001	1.28	2.81
Cirrhosis	1.49	0.43	1.38	0.167	0.85	2.63
Haematemesis	1.96	0.42	3.16	0.002	1.29	2.98
Transfusions number	1.11	0.03	3.48	0.000	1.05	1.18
Timing to endoscopy interaction						
Transfusion before after endoscopy						
0/6 hours Before endoscopy	1.06	0.25	0.26	0.795	0.67	1.69
6/12 hours After endoscopy	1.06	0.35	0.18	0.858	0.55	2.04
6/12 hours Before endoscopy	0.49	0.22	-1.59	0.112	0.20	1.18
12/24 hours After endoscopy	0.78	0.33	-0.58	0.562	0.34	1.79
12/24 hours Before endoscopy	0.92	0.36	-0.22	0.826	0.42	1.99
>24 hours After endoscopy	1.13	0.53	0.26	0.799	0.45	2.85
>24 hours Before endoscopy	0.95	0.56	-0.09	0.928	0.30	3.00
Admission_Haemoglobin, g/dL						
<7	Reference					

7-8	1.14	0.28	0.54	0.589	0.70	1.86
8-10	1.02	0.25	0.10	0.919	0.64	1.64
>10	0.82	0.26	-0.62	0.534	0.43	1.54
Source of bleeding	0.86	0.27	-0.48	0.632	0.46	1.59
_cons	0.00	0.00	-10.53	0.000	0.00	0.00

Supplementary Figure S1. Adjusted death risk, including source of bleeding, by interaction of haemoglobin value at admission and red pack cell transfusion before or after endoscopy

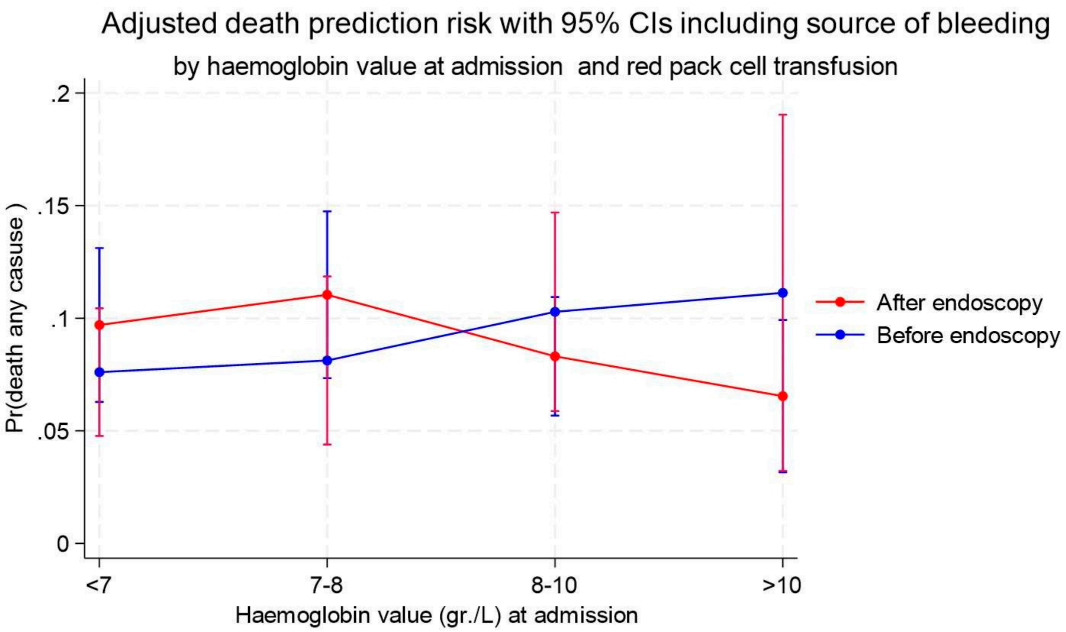

Supplementary Figure S2. Adjusted death risk, including source of bleeding, by interaction of red pack cell transfusion before or after endoscopy and timing to endoscopy

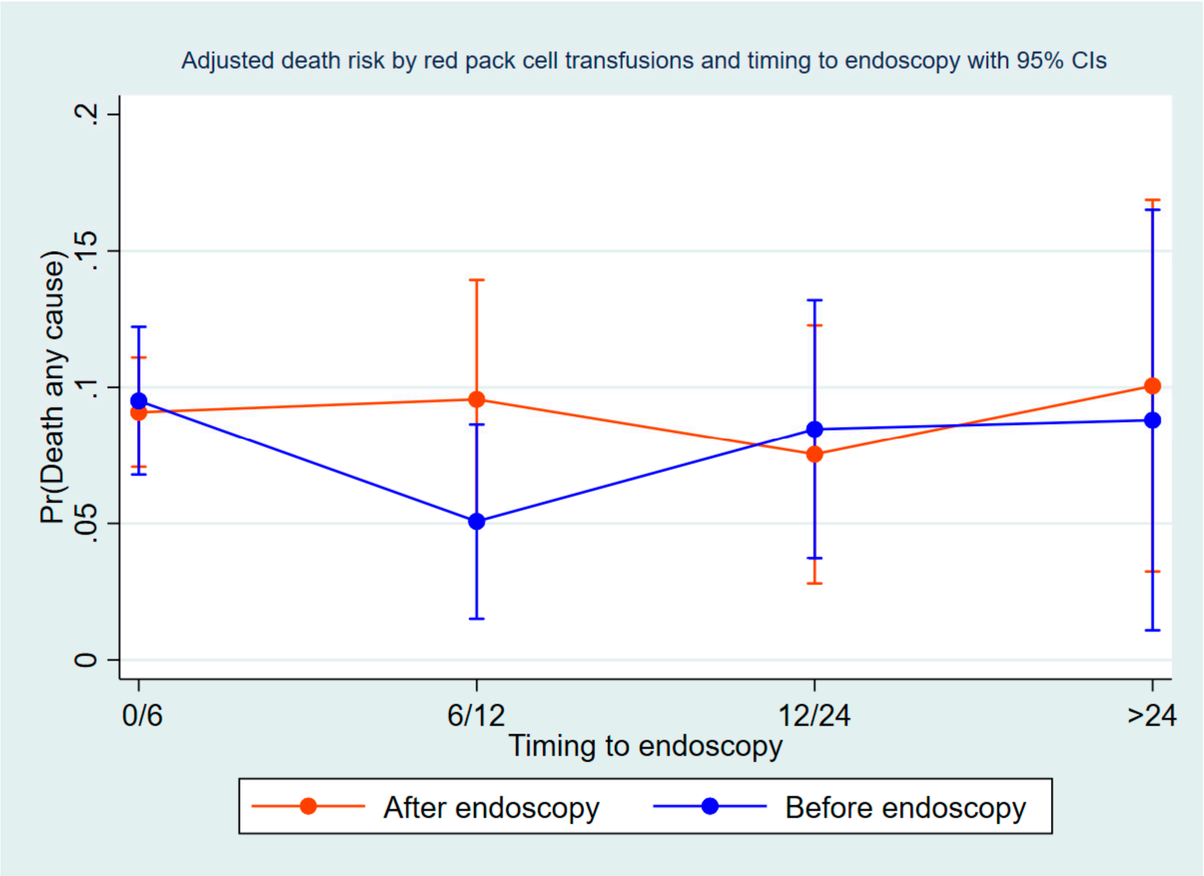

Supplement: Supplementary file 1 [file diseases-13-00329-s001.zip › diseases-3833938-supplementary.pdf]
